# How is edaravone effective against acute ischemic stroke and amyotrophic lateral sclerosis?

**DOI:** 10.3164/jcbn.17-62

**Published:** 2017-11-11

**Authors:** Kazutoshi Watanabe, Masahiko Tanaka, Satoshi Yuki, Manabu Hirai, Yorihiro Yamamoto

**Affiliations:** 1 Sohyaku. Innovative Research Division, Mitsubishi Tanabe Pharma Corporation, 1000 Kamoshida-cho, Aoba-ku, Yokohama 227-0033, Japan; 2 School of Bioscience and Biotechnology, Tokyo University of Technology, 1404-1 Katakura-cho, Hachioji 192-0982, Japan; 3 Ikuyaku. Integrated Value Development Division, Mitsubishi Tanabe Pharma Corporation, 17-10 Nihonbashi-Koamicho, Chuo-ku, Tokyo 103-8405, Japan; 4 Ikuyaku. Integrated Value Development Division, Mitsubishi Tanabe Pharma Corporation, 3-2-10 Dosho-machi, Chuo-ku, Osaka 541-8505, Japan

**Keywords:** edaravone, radical scavenger, antioxidant, acute ischemic stroke, amyotrophic lateral sclerosis

## Abstract

Edaravone is a low-molecular-weight antioxidant drug targeting peroxyl radicals among many types of reactive oxygen species. Because of its amphiphilicity, it scavenges both lipid- and water-soluble peroxyl radicals by donating an electron to the radical. Thus, it inhibits the oxidation of lipids by scavenging chain-initiating water-soluble peroxyl radicals and chain-carrying lipid peroxyl radicals. In 2001, it was approved in Japan as a drug to treat acute-phase cerebral infarction, and then in 2015 it was approved for amyotrophic lateral sclerosis (ALS). In 2017, the U.S. Food and Drug Administration also approved edaravone for treatment of patients with ALS. Its mechanism of action was inferred to be scavenging of peroxynitrite. In this review, we focus on the radical-scavenging characteristics of edaravone in comparison with some other antioxidants that have been studied in clinical trials, and we summarize its pharmacological action and clinical efficacy in patients with acute cerebral infarction and ALS.

## Importance of peroxyl radical scavenger

Reactive oxygen and nitrogen species such as superoxide anion radical (O_2_^•−^), hydrogen peroxide, hydroxyl radical, singlet oxygen, peroxyl radicals, hypochlorous acid, and peroxynitrite play roles in the pathogenesis and exacerbation of various diseases, and therefore drugs that scavenge reactive oxygen species may have therapeutic value. On the other hand, superoxide anion radical and hydrogen peroxide may not be promising targets for drug development, since they are scavenged by endogenous enzymes.^(1)^ Further, singlet oxygen has a short lifespan and does not cause chain oxidation.^(1,2)^ Hydroxyl radical react with all organic compounds in a diffusion-limited manner,^(1)^ and thus are also not a suitable drug target. However, when hydroxyl radical is generated in the body, they react with lipids, proteins, and carbohydrates—which are abundant components of the body—and the resulting carbon radicals are converted to peroxyl radicals by addition of oxygen.^(3)^ Peroxyl radicals are much less reactive than hydroxyl radical, and consequently have much longer lifespans.^(3)^ Therefore, drugs that scavenge peroxyl radicals were initially chosen as candidates for development. α-Tocopherol (vitamin E) and reduced coenzyme Q10 are the most important scavengers of lipid-soluble peroxyl radicals,^(4,5)^ and ascorbic acid (vitamin C) is an important scavenger of water-soluble peroxyl radicals.^(6)^

Among the principal components of the body, lipids, including polyunsaturated fatty acids, are the most easily oxidized.^(3,7)^ Oxidation of lipids proceeds as a chain reaction^(7,8)^ in which a single radical can generate thousands of molecules of lipid hydroperoxide, causing damage to biological membranes and leading to functional impairment;^(9)^ therefore, it is especially important to suppress such oxidation. Since lipid peroxyl radicals play the main role in the chain reaction, development of drugs to scavenge lipid peroxyl radicals is considered a priority.

Under pathological conditions, such as ischemia-reperfusion and inflammation, production of reactive oxygen species *in vivo* is dramatically increased.^(1)^ Large amounts of nitric oxide are produced in inflammatory tissues, and nitric oxide combines with superoxide anion radical to produce peroxynitrite.^(10)^ Peroxynitrite nitrates tyrosine residues in proteins, leading to abnormal aggregation of the proteins or aberrant enzyme activities.^(11)^ Although uric acid is an endogenous scavenger of peroxynitrite,^(10)^ its activity is insufficient, and this is another reason why peroxynitrite is an attractive target for drug development.

## Compound design and selection of edaravone

There is extensive evidence that free radicals play an important role in injury after cerebral ischemia. Ischemia alone, or ischemia followed by reperfusion, leads to increased production of free radicals, which attack cell membranes by peroxidizing unsaturated fatty acids in phospholipids. The resulting chain reaction leads to ischemic brain injury, manifested as edema, infarction, and neuronopathy.^(12–14)^

**Fig. 1:**
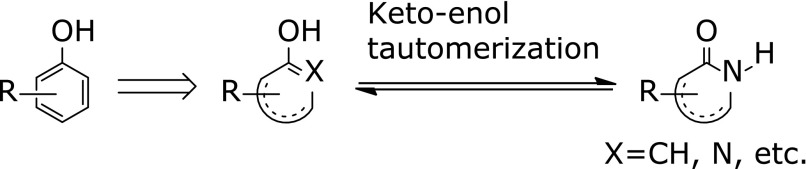
Design of a phenol-like compound.

In 1984, Mitsubishi Yuka Pharmaceutical Corporation started a research program to develop free radical-scavenging agents based on the concept that free radical scavengers could play a role in the treatment of acute cerebral infarction. Phenol derivatives are widely known to have radical-scavenging activity, they are usually not suitable for use as drugs because they are generally irritating, corrosive and highly toxic. Noting that a hydroxy group directly connected to an aromatic benzene ring is the key structure for radical-scavenging activity of phenol derivatives, the researchers thought that aromatic heterocycles in which a hydroxy group would be generated by keto-enol tautomerization should have radical-scavenging activity comparable to that of phenol. In other words, recognizing that the carbonyl group of heterocyclic amides or ketones could be converted to a hydroxy group by enolization, the researchers thought that a cyclic amide or a ketone moiety in a heterocyclic ring would be equivalent to an aromatic ring with a hydroxy group owing to enolization, and thus such compounds might have similar activity to phenol derivatives (Fig. 1). Based on this idea, the researchers designed and synthesized a variety of compounds, and edaravone (3-methyl-1-phenyl-2-pyrazolin-5-one) was identified as an active compound.

**Fig. 2:**
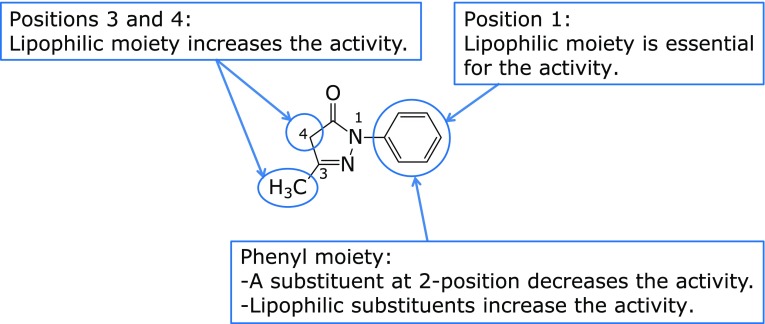
Optimization of 3-methyl-1-phenyl-2-pyrazolin-5-one.

Using edaravone as the lead compound, the researchers set out to optimize the substituents for *in vitro* lipid peroxidation-inhibitory activity in rat brain homogenate.^(15)^ Introduction of a hydrophilic or polar group at any position on the 2-pyrazolin-5-one ring resulted in decreased lipid peroxidation-inhibitory activity, whereas introduction of a lipophilic substituent on the ring maintained or increased the activity. There was no clear relationship between the electronic properties of the substituents and the activity (Fig. 2). Notably, the derivative with two methyl groups at the 4-position had no activity to inhibit lipid peroxidation, presumably because keto-enol tautomerization was blocked. This observation supported the design hypothesis described above.

Compounds with *in vitro* lipid peroxidation-inhibitory activity were assessed by evaluating electroencephalographic recovery and prolongation of survival in a rat model of whole-brain ischemia (10 min)-reperfusion (10 mg/kg, i.d., administered 30 min before ischemia induction).^(15)^ Edaravone and eight other compounds showed activity in this model, and edaravone was selected as a candidate for further development after evaluation of these compounds in a rat cerebral edema model and toxicity studies.^(16)^ Edaravone was developed as an injection, because the target patients often have impaired consciousness and dysphagia.

## History of development

In 1987, Mitsubishi Chemical Industries (incorporating Mitsubishi Yuka Pharmaceutical Corporation) started clinical studies of edaravone under the code name MCI-186, and in 2001, Mitsubishi Tokyo Pharmaceuticals Inc. (formed by a merger between Mitsubishi Chemical Corporation and Tokyo Tanabe Co., Ltd.) obtained manufacturing approval for edaravone (Radicut^®^ injection) for the indications of neurological symptoms, impaired activities of daily living, and functional impairment due to acute cerebral infarction, with the condition that administration be initiated within 24 h of onset. Unlike existing anticoagulants and antiplatelet agents, edaravone can be used not only in cerebral thrombosis, but also in cerebral embolism, and it is the first drug in Japan whose efficacy has been confirmed by assessment of dysfunction according to the modified Rankin scale, which is used internationally.

On the other hand, amyotrophic lateral sclerosis (ALS) is a neurodegenerative disease in which upper and lower motor neurons sporadically and progressively degenerate and are lost, and generalized muscle atrophy and muscle weakness occur. The median time from onset of ALS to death or to the need for invasive ventilation has been reported to be 20 to 48 months.

Various factors have been reported to be involved in the onset and progression of ALS, and one of them is considered to be oxidative stress induced by free radicals. Based on this information and the results of a study of edaravone in wobbler mice.^(17)^ Mitsubishi Tokyo Pharmaceutical Inc. began clinical studies of edaravone in ALS in 2001. In 2005, edaravone was designated as an orphan drug with ALS as the expected indication or target disease. After one Phase 2 study and two pivotal Phase 3 studies, Mitsubishi Tanabe Pharma Corporation obtained marketing approval in Japan in 2015 for the use of edaravone to inhibit progression of dysfunction in ALS. In 2017, the U.S. Food and Drug Administration also approved edaravone for the same purpose.

This review focuses on the radical-scavenging and antioxidant activities of edaravone, and summarizes its pharmacological action and the results of clinical studies in patients with acute cerebral infarction and ALS.

## Properties of Edaravone

### Physicochemical properties

**Fig. 3:**
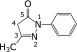
Chemical structure of edaravone.

Edaravone is a 2-pyrazolin-5-one derivative bearing a phenyl group at the 1-position and a methyl group at the 3-position (Fig. 3). It was synthesized in 1883 by Knorr, via the reaction of phenylhydrazine and acetoacetic ester.^(18)^

The physiochemical properties of edaravone are summarized in Table 1. The acid dissociation constant (pKa) of edaravone is 7.0 and edaravone has the properties of a weak acid, dissociating to afford the anion with release of a proton. Thus, the solubility of edaravone in water is pH-dependent, being roughly constant from pH 2 to 7, and then increasing from pH 8 to 10.^(19)^ In water/1-octanol, partitioning to water also increases with pH. In water at pH 7.4, the percentages of the neutral and anionic forms of edaravone have been calculated to be 28.5% and 71.5%, respectively.^(20)^

**Fig. 4:**
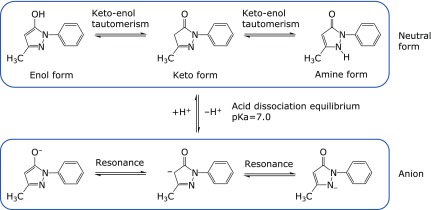
Edaravone tautomerism and acid dissociation equilibrium.

Edaravone also exhibits keto-enol tautomerism (Fig. 4), and has three isomeric structures: the keto form shown in Fig. 3, an enol form with a hydroxy group at the 5-position (phenol-like structure), and an amine form with a hydrogen atom on the nitrogen atom at the 2-position. Like the neutral molecule, the edaravone anion also possesses three resonance structures. Thus, under physiological conditions (in water, pH 7.4), edaravone exists as a mixture of its neutral and anionic forms, with each having tautomeric forms and resonance structures. The equilibrium between neutral and anionic forms of edaravone and the presence of keto-enol tautomerism have an important effect on the radical-scavenging activity of edaravone, which is the basis of the antioxidant activity of edaravone in the body, as will be discussed later.

### Radical scavenging and antioxidant activities

#### Oxidation potential

Two groups^(21,22)^ have measured the oxidation potential of edaravone (Table 2). The oxidation potential decreases as the pH (namely, the proportion of anions) increases.

#### Reaction of edaravone with various radicals

Reactions of edaravone with various radicals have been studied. Radical-scavenging activity towards the stable radical, 1,1-diphenyl-2-picrylhydrazyl (DPPH), was determined by electron spin resonance (ESR), and edaravone almost completely eliminated the ESR signal when added to DPPH solution.^(23)^

Rate constants between edaravone and several radicals have been reported (Table 3).^(24–26)^ The observed rate constants between edaravone and every radical except the aryl radical are diffusion-limited, or nearly so, and edaravone has excellent radical-scavenging activity against the hydroxyl radical, trichloromethylperoxyl radical and other studied radicals. The only exception is the aryl radical, presumably due to steric hindrance around the radical. Reaction rates of edaravone with alkoxyl radicals and peroxyl radicals (in H_2_O, single electron transfer) have also been calculated,^(27)^ and all of the reaction rate constants are 10^7^ M^−1^s^−1^ or higher, indicating that edaravone would also be a good scavenger of these radicals.

The reaction of edaravone with superoxide anion radical produced by xanthine-xanthine oxidase was studied by the method of Butler *et al.*^(28)^ It was observed that 100 µM edaravone showed no reactivity with superoxide anion radical.^(29)^

To compare edaravone with other representative radical scavengers, Kamogawa and Sueishi^(30)^ studied the relative reaction rates of the radical-scavenging reactions of edaravone and other radical scavengers against multiple simultaneously generated radical species (hydroxyl radical, superoxide anion radical, alkoxyl radical, *tert*-butylperoxyl radical, methyl radical, singlet oxygen) by photolysis reaction, using the ESR trapping technique. This study revealed that edaravone had radical-scavenging activity similar to that of trolox against singlet oxygen, hydroxyl radical, superoxide anion radical, and alkoxyl radical, and it had a higher radical-scavenging activity than uric acid or glutathione towards hydroxyl radical, *tert*-butylperoxyl radical, and methyl radical. Whereas uric acid, glutathione, and trolox showed selectivity towards certain radical species, edaravone showed high radical scavenging activity against all of the radical species studied (Table 4). In ischemia-reperfusion, it is thought that hydroxyl radical and superoxide anion radical react with a great variety of molecules in the body to generate many types of secondary radical species, especially from lipids. In this context, the broad scavenging activity of edaravone is expected to be effective in counteracting pathological processes.

In short, the above studies showed that edaravone has excellent radical-scavenging activity against a wide range of oxidative radical species.

#### Activity of edaravone against lipid peroxidation

 Edaravone concentration-dependently inhibited the formation of linoleic acid hydroperoxide by hydroxyl radical generated via the Fenton reaction in a hydrogen peroxide-water and iron (II) ion system (IC_25_ = 33.8 µM against 100 µM linoleic acid).^(29)^ Edaravone also concentration-dependently inhibited lipid peroxidation induced by incubation (37°C, 30 min) of brain homogenate prepared from rat cerebrum (IC_50_ = 15.3 µM).^(29)^ These results show that edaravone inhibits lipid peroxidation involving radical chain reactions, and has lipid peroxidation-inhibitory activity in biological systems.

**Fig. 5:**
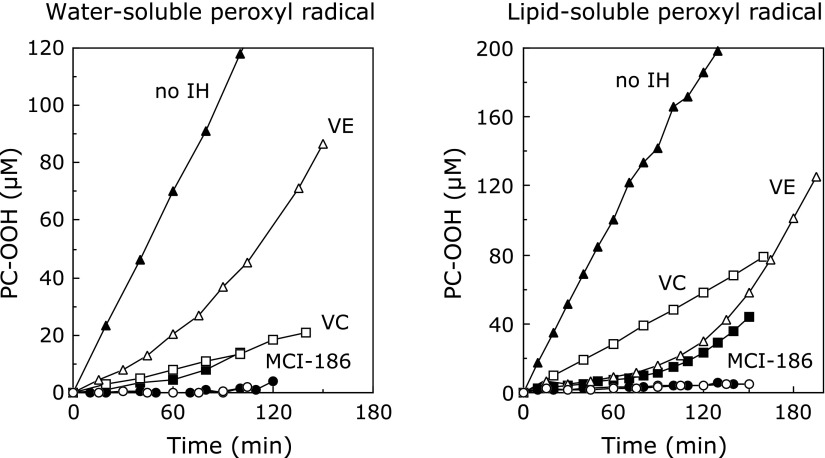
Effects of edaravone on peroxidation of liposome membranes by water- and lipid-soluble free radicals. PC-OOH: phosphatidylcholine hydroperoxide; ▲: without antioxidant; □: ascorbic acid (100 µM); ■: edaravone (50 µM); ◯: edaravone (50 µM) + α-tocopherol (2 µM); △: α-tocopherol (2 µM); ●: edaravone (50 µM) + ascorbic acid (100 µM). (Reprinted from ref 31: ‘Antioxidant activity of 3-methyl-1-phenyl-2-pyrazolin-5-one’, Yamamoto Y, Kuwahara T, Watanabe K, Watanabe K., Redox Report, 1996, 2, pp. 333–338, Taylor & Francis Ltd., reprinted by permission of the publisher Taylor & Francis Ltd. http://www.tandfonline.com)

The effect of edaravone on lipid peroxidation of artificial phospholipid liposome membranes by water- or lipid-soluble radicals has been investigated. Yamamoto *et al.*^(31)^ induced peroxidation of liposome membranes by water- and lipid-soluble peroxyl radicals generated by water- and lipid-soluble azo compounds, and quantified the time course of phosphatidylcholine hydroperoxide formation in the presence of various antioxidants (Fig. 5). Ascorbic acid showed potent antioxidant activity against water-soluble radicals, and α-tocopherol showed potent antioxidant activity against lipid-soluble radicals, while edaravone had similarly potent inhibitory effects on lipid peroxidation by both kinds of radicals. Lipid peroxidation proceeds as a chain reaction through generation of a lipid peroxyl radical by abstraction of a hydrogen atom from the methylene group adjacent to a double bond in a lipid molecule, followed by addition of an oxygen molecule, and the resulting lipid peroxyl radical abstracts a hydrogen atom from another lipid molecule and thereby initiates a new reaction cycle. Edaravone inhibits the lipid peroxidation chain reaction by scavenging peroxyl radicals responsible for the reaction. Furthermore, addition of ascorbic acid or α-tocopherol increased the antioxidant activity of edaravone, and peroxidation of phosphatidylcholine was almost completely blocked. Based on these results, it is expected that edaravone would effectively inhibit lipid peroxidation in cell membranes of the body in cooperation with ascorbic acid and α-tocopherol, which are endogenous antioxidants. Ohara *et al.*^(26)^ showed that edaravone had good antioxidant activity against α-tocopherol radical generated by radical scavenging, which suggests that edaravone may regenerate α-tocopherol in the body. Thus, edaravone may exert antioxidant activity not only by directly scavenging radicals, but also by acting in concert with α-tocopherol and other antioxidants already present in the body.

Inhibitory activity against oxidative damage to cells was reported to be based on the inhibitory effect of edaravone on lipid peroxidation. Edaravone showed concentration-dependent inhibition of cultured bovine aortic endothelial cell damage induced by 15-hydroperoxy-eicosatetraenoic acid (15-HPETE) (30 µM), and 1 µM edaravone inhibited cell death by 57% as compared with the control group.^(32)^ HPETE is a hydroperoxide produced via an arachidonic acid metabolic pathway that is activated in cerebral ischemia-reperfusion, with formation of hydroxyl radical. Infusion of arachidonic acid into rat brain also induced oxidative damage via HPETE formation, leading to cerebral edema. Edaravone strongly inhibited cerebral edema in this model.^(32)^

### Mechanisms of radical scavenging by edaravone

#### Effect of pH on antioxidant activity of edaravone

 Yamamoto *et al.*^(31)^ investigated the effect of pH on scavenging by edaravone of peroxyl radicals generated by thermal decomposition of azo compounds in methanol or methanol/buffer at various pH values. The rate of edaravone consumption increased with increasing pH. As discussed above, the pKa of edaravone is 7.0, and approximately a half of edaravone exists as edaravone anions at physiological pH. The rate of generation of peroxyl radicals from azo compounds is thought to be independent of solvent pH. Thus, the anionic form of edaravone may be more reactive than the neutral form.

Ohara *et al.*^(26)^ measured the secondary reaction rate constant of the reaction between edaravone and 2,5-di-*tert*-butyl-4-(4-methoxyphenyl)phenoxyl (ArO^•^) in water-methanol (1/1, v/v) at various pH values. The reaction rate increased with increasing pH; at pH >6 to <8, at which ArO^•^ is stable, the secondary reaction rate constant increased from 6.28 × 10^1^ M^−1^s^−1^ at pH 6 to 1.08 × 10^2^ M^−1^s^−1^ at pH 8 (Table 3). These results confirm that edaravone has greater radical-scavenging activity at higher pH, supporting the idea that the anionic form of edaravone has higher activity.

#### Reaction products of edaravone with radicals

Yamamoto *et al.*^(31)^ studied the time-course of formation of the reaction products of edaravone and alkyl peroxyl radicals generated by thermal decomposition of azo compounds, as well as the nature of the products. Edaravone was rapidly consumed. Initially, 3-methyl-1-phenyl-2-pyrazolin-4,5-dione (4-oxoedaravone) was observed as the main product, along with small amounts of 4-hydroxy-4-(3-methyl-5-oxo-1-phenyl-2-pyrazolin-4-yl)-3-methyl-1-phenyl-2-pyrazolin-5-one (BPOH). As BPOH disappeared, 4-oxoedaravone began to decrease, and 2-oxo-3-(phenylhydrazono)butanoic acid (OPB) gradually increased. The chemical structure of BPOH suggested that the intermediate BPOH was generated by nucleophilic addition reaction of edaravone with 4-oxoedaravone in equilibrium. This is consistent with the fact that BPOH was observed only in the presence of both edaravone and 4-oxoedaravone, and that formation of BPOH was not observed after consumption of edaravone. The chemical structure of OPB indicates that OPB is a hydrolysis product of 4-oxoedaravone, and transformation of 4-oxoedaravone to OPB was independent of the existence of radicals; therefore, OPB appears to be generated by hydrolysis of 4-oxoedaravone by water used as the solvent. Furthermore, no adducts of edaravone with radicals were observed as reaction products. Formation of BPOH and OPB as reaction products was also observed in the reaction between edaravone and hydroxyl radical or DPPH.^(23)^

Formation of OPB was also observed in the reaction of edaravone with alkyl peroxyl radicals generated from methyl linoleate hydroperoxide and iron (III) ion. Adducts of edaravone with radicals were not observed, and no radicals were generated when these reactions were performed in the absence of iron (III) ion. This indicates that edaravone reacts with radicals, but not with hydroperoxide.^(23)^ A trimer, [4,4-bis-(3-methyl-5-oxo-1-phenyl-2-pyrazolin-4-yl)-3-methyl-1-phenyl-2-pyrazolin-5-one, TP], was obtained as the product when edaravone and DPPH were allowed to react in the absence of oxygen.^(23)^

#### Radical scavenging mechanism of edaravone

**Fig. 6:**
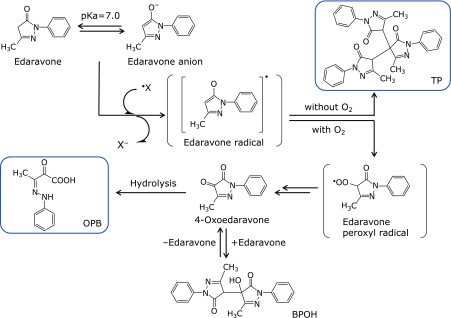
A hypothetical mechanism for the production of edaravone radical by electron transfer from edaravone anion to peroxyl radical and the formation of oxidation products.

The time-course studies and the chemical structures of the reaction products described above indicate that the reaction pathway of edaravone with radicals is as shown in Fig. 6. Edaravone anion donates one electron to a radical, converting the radical to the corresponding anion. The resulting edaravone radical is converted to an edaravone peroxyl radical by reaction with an oxygen molecule; then the edaravone peroxyl radical is converted to OPB via 4-oxoedaravone. In the absence of oxygen, edaravone peroxyl radical are not generated, but react with each other, generating TP. Thus, OPB is the reaction product of radical scavenging by edaravone under an oxygen-containing atmosphere regardless of the radical species. Since edaravone is metabolized into the corresponding glucuronide and sulfate conjugates, generation of OPB indicates that radical scavenging has occurred.

Edaravone does not react with the superoxide anion radical, as described above. The reason for this may be electrostatic repulsion between the edaravone anion and the superoxide anion radical.

Ono *et al.*^(33)^ investigated the reactivity of edaravone-derived radical species by means of density functional calculations, using 1,3-dimethyl-2-pyrazolin-5-one (in which the phenyl group at the 1-position of edaravone is replaced with a methyl group) as a model compound. The unpaired electron of edaravone radical is highly delocalized, and consequently, the edaravone radical should be less reactive than reactive oxygen radicals such as hydroxyl radical, alkyl peroxyl radicals, and alkoxyl radicals, which cause lipid peroxidation and subsequent radical chain reactions. Calculations of the model compound indicated that the pyrazoline radical was stabilized by delocalization of the unpaired electron, and the spin density of the unpaired electron was highest on the nitrogen atom at the 2-position, the carbon atom at the 4-position, and the oxygen atom bound to the carbon atom at the 5-position. The singly occupied molecular orbital (SOMO) of the pyrazoline radical or pyrazolin-peroxyl radical lay at higher energy than those of the hydroxyl radical, methoxyl radical, and methyl peroxyl radical. Therefore, the reactivity of the pyrazoline radical and pyrazolin-peroxyl radical should be much less than that of these reactive radicals. These results indicate that the radical species derived from edaravone is relatively stable and would not generate lipid radicals by reacting with lipids or induce lipid peroxidation by radical chain reaction, as reactive oxygen radicals do. Ono *et al.*^(33)^ also suggested that the preferred outcome of radical scavenging by edaravone was likely to be OPB generation. Hata *et al.*^(34)^ reported that the radical scavenging mechanism of 1,3-dimethyl-2-pyrazolin-5-one was electron transfer from its anion to the radicals, based on studies of the absorption spectra of the intermediates in reactions between 1,3-dimethyl-2-pyrazolin-5-one, and hydroxyl radical and azide radical (N_3_^•^).Thus, edaravone has radical scavenging activity against a variety of radicals, and its activity can be attributed not to formation of adducts with the radicals, but rather to scavenging by electron donation, and the product of the scavenging reactions is OPB. These conclusions are consistent with the fact that adducts with edaravone and radical species were not observed in the reactions with various radicals.

Wang and Zhang^(35)^ investigated the radical-scavenging mechanism of edaravone against DPPH by means of density functional theory calculation of bond dissociation enthalpies and ionization potentials. They inferred that the radical scavenging mechanism of edaravone in lipid systems was hydrogen atom abstraction and that the hydrogen atom at the 4-position of edaravone was donated, as it has a low carbon-hydrogen bond dissociation energy.

#### Discussion of radical-scavenging activity

Nakagawa *et al.*^(21)^ synthesized edaravone derivatives and evaluated their oxidation potential and radical-scavenging activity against hydroxyl radical. They measured radical-scavenging activity in terms of the inhibitory activity of the derivatives on formation of adducts of 5,5-dimethyl-1-pyrroline-*N*-oxide (DMPO) and hydroxyl radical generated by UV irradiation of hydrogen peroxide. They concluded that the amount of anions in the reaction system was important, because edaravone and its derivatives scavenged radicals by one-electron transfer from their anions. The oxidation potential of the pyrazolin-5-one ring was decreased in derivatives with electron-donating substituents, and the proportion of the anion was decreased due to the increase in negative charge; in contrast, the proportion of the anionic form was increased for derivatives with electron-withdrawing substituents, and the oxidation potential was also increased. In the case of edaravone, the oxidation potential and the anion proportion were well balanced, and as a result, edaravone had the highest radical-scavenging activity among the derivatives studied. Similarly, Pérez-González and Galano^(36)^ calculated the ionization energies, proton affinities, and pKa values of edaravone derivatives with substituents introduced at the 1-position phenyl group and at the 3-position of pyrazolin-5-one, and compared the free radical-scavenging activity of the derivatives via the single-electron transfer mechanism. With the derivatives possessing electron-withdrawing groups, proton affinity decreased and the amount of anion increased, and electron-donating activity and antioxidant activity by single-electron transfer decreased because the ionization energy became higher than that of edaravone. In contrast, with the derivatives possessing electron-donating groups, proton affinity increased and the amount of anion decreased, while the ionization energy became lower than that of edaravone, and electron-donating activity increased. Thus, as discussed by Nakagawa *et al.*^(21)^ and Pérez-González and Galano, the balance of the ionization energy (which is related to the electron-donating activity) and the amount of the anion (responsible for single-electron transfer) is important for radical-scavenging activity, and the two are well-balanced in the case of edaravone.

It is important to note that Nakagawa *et al.*^(21)^, and Pérez-González and Galano^(36)^ studied radical-scavenging activity in aqueous solutions; however, lipophilic environments are also important under physiological conditions. As the pKa of edaravone is 7.0, the neutral molecule accounts for 28.5% of edaravone under physiological conditions (pH 7.4),^(20)^ and so edaravone should be able to enter lipid environments such as cell membranes, and scavenge peroxyl radicals generated from lipids in radical chain reactions. Thus, in summary, edaravone has the following features:^(26)^

- Its pKa (7.0) is close to the physiological pH (7.4), and edaravone exists as both a neutral molecule and an anion in the body.
- Edaravone anion exhibits potent radical-scavenging activity via an electron-donating mechanism.
- Edaravone can react with a wide variety of radical species.
- Edaravone can readily enter biological membranes by passive diffusion [the Caco-2 cell permeability of edaravone is similar to that of propranolol (unpublished data)].
- Consequently, edaravone is distributed to both aqueous parts of the body and hydrophobic parts, such as biological membranes: in other words, to both plasma and tissues.
- Since edaravone exerts radical-scavenging activity in both aqueous and lipophilic environments, it is an effective antioxidant.
- Edaravone exerts its antioxidant activity in concert with other antioxidants in the body, including ascorbic acid and α-tocopherol.

Thus, edaravone is well distributed to both the aqueous phase where radicals are primarily generated, and the lipid phase where lipid peroxidation proceeds by radical chain reaction via secondarily induced peroxyl radicals, and it should scavenge radicals in both environments.

### Effects of edaravone on peroxynitrite

**Scheme 1:**
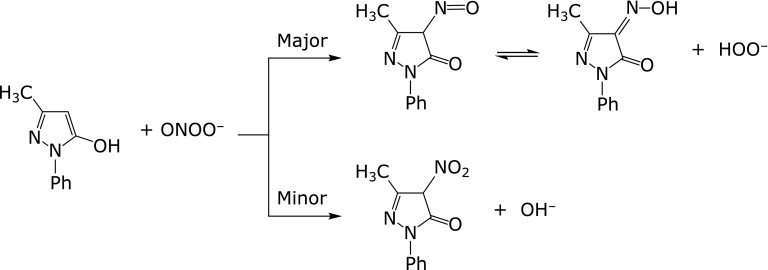


Fujisawa and Yamamoto^(37)^ investigated the reaction of edaravone with peroxynitrite. Reaction with edaravone and 3-(4-morpholinyl)sydnonimine hydrochloride (SIN-1), a peroxynitrite donor, in phosphate buffer resulted in formation of 3-methyl-4-nitroso-1-phenyl-pyrazolin-5-one (4-NO-edaravone) as the major product and 3-methyl-4-nitro-1-phenyl-pyrazolin-5-one (4-NO_2_-edaravone) as a minor product (Scheme 1). Hydroxyl radical and/or nitrogen dioxide, which are generated by the decomposition of peroxynitrite, were not formed to any great extent in this reaction.

Fujisawa and Yamamoto^(37)^ also investigated the competitive reactions of SIN-1 with edaravone and uric acid (an endogenous peroxynitrite scavenger). Decrease of edaravone was much faster than that of uric acid, and the reactivity of peroxynitrite with edaravone was nearly 30 times larger than that with uric acid. The observed reaction rate constant of edaravone with peroxynitrite was 1.5 × 10^4^ M^−1^s^−1^, calculated from the above result and the reported reaction rate constant of uric acid and peroxynitrite.

The effects of edaravone on peroxynitrite-induced oxidative damage were also investigated.^(38)^ SIN-1 induced nerve cell death in a dose- and time-dependent manner in aqueous solution; the survival rate of cultured nerve cells was 35% after treatment with 500 µM SIN-1 for 24 h. This was significantly increased to 51% and 65%, respectively, in the presence of 10 and 100 µM edaravone. Oxidative stress by SIN-1 was dendrotoxic, and dendritic beading was observed in the dendritic branches. Treatment with SIN-1 (500 µM) induced dendritic beading in 30% of the nerve cells that survived, and edaravone significantly decreased the number of cells in which beading was formed (by 26% and 18% at 10 and 100 µM, respectively). The number of beads per nerve cell was also significantly decreased.

## *In vivo* radical scavenging and antioxidant activity

### Effects in animal models

**Fig. 7:**
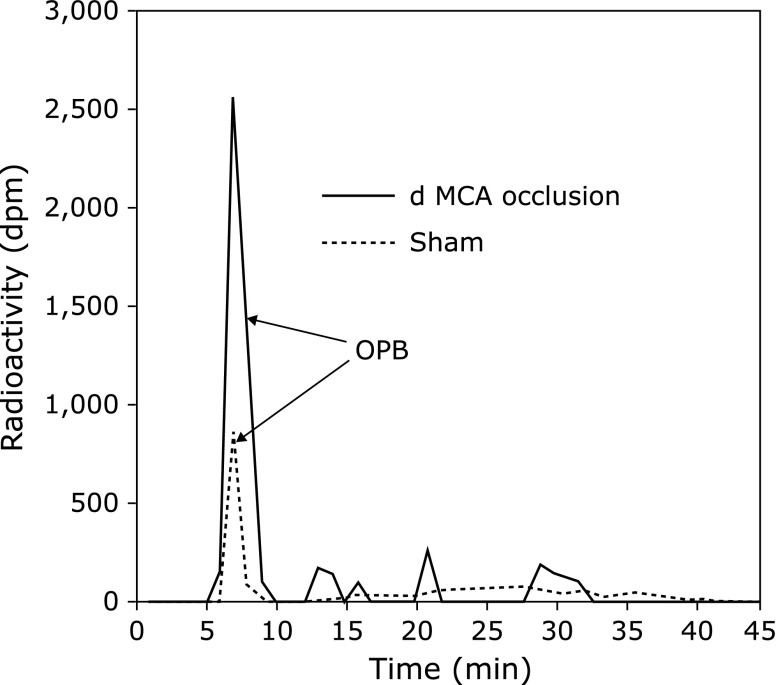
Typical chromatogram of extracts from the rat cerebral cortex after perfusion of ^14^C-edaravone. (Reprinted from ref 39: ‘Effects of a novel free radical scavenger, MCI-186, on ischemic brain damage in the rat distal middle cerebral artery occlusion model’, J Pharmacol Exp Ther, Kawai H, Nakai H, Suga M, Yuki S, Watanabe T, Saito K, 1997, 281(2), pp. 921–927, reprinted by permission of The American Society for Pharmacology and Experimental Therapeutics, http://www.aspet.org)

Kawai *et al.*^(39)^ detected OPB, the reaction product of edaravone radical scavenging, in a rat photothrombotic distal middle cerebral artery (MCA) occlusion model. ^14^C-labeled edaravone was perfused during ischemia through a microdialysis probe implanted in a motor area of the cortex in the penumbra region. The brain was removed 90 min after ischemia and the levels of OPB were measured in homogenate of the ipsilateral cerebral cortex. The OPB level in the ischemia group was significantly higher than that in the sham group (Fig. 7). This result strongly suggests that free radical scavenging by edaravone occurred *in vivo*. Edaravone also significantly reduced the infarct volume and ameliorated neurological symptoms in the same model. These results indicate that edaravone protects the brain by scavenging radicals in the region of the ischemic penumbra.

Yamamoto *et al.*^(40)^ investigated the antioxidant activity of edaravone *in vivo* using monounsaturated fatty acids in plasma as a marker of oxidative brain ischemia-reperfusion injury. Monounsaturated fatty acids in plasma are an effective marker of oxidative damage associated with radicals; for example, in a carbon tetrachloride-induced hepatic injury model in rats^(41)^ and a metal-accumulating Long-Evans Cinnamon rat model.^(42)^ Yamamoto *et al.*^(40)^ investigated oxidative stress injury and the effects of edaravone in cerebral infarction using a rat MCA 2-h occlusion-reperfusion model by monitoring changes in the percent ratios (with respect to total free fatty acids) of palmitoleic acid (16:1) and oleic acid (18:1) in plasma (%16:1 and %18:1, respectively). The %16:1 was significantly increased between Days 1 and 5 after MCA occlusion-reperfusion, and %18:1 was significantly increased between Days 1 and 7 and at Day 14. These results can be explained in terms of an imbalance in the *in vivo* oxidation-prevention mechanisms both inside and around the cells, resulting in the oxidation of mainly polyunsaturated fatty acids in cell membranes, since these are the most easily oxidized fatty acids. Fatty acid desaturase is activated in cells to compensate for the decrease in polyunsaturated fatty acids, and transforms stearic acid (18:0) and palmitic acid (16:0) to 18:1 and 16:1, respectively. Free fatty acids in the cells are then observed in plasma due to the actions of hydrolytic enzymes after cell death caused by further increase of oxidative stress. The rat MCA occlusion-reperfusion model employed in this study is widely used, and oxidative injury by active oxygen species plays an important role in the pathology. Thus, the increase in monounsaturated fatty acids that was observed in this study is thought to reflect increased oxidative injury in the brain.

Yamamoto *et al.*^(40)^ also investigated the effects on %16:1 and %18:1 of edaravone 3 mg/kg administered twice a day for 14 days immediately after MCA occlusion-reperfusion. The increase in %16:1 was significantly suppressed between Day 1 and Day 14 following MCA occlusion-reperfusion in the group that received edaravone, and the increase in %18:1 was significantly suppressed between Day 1 and Day 10. The effects of repeated administration of edaravone were also investigated in comparison with a single-dose administration group that received edaravone only immediately after MCA occlusion-reperfusion. Although the increase in %16:1 was significantly inhibited between Day 1 and Day 7, and at Day 14, the increase in %18:1 was not inhibited at any of these time points in the single-dose administration group. The above results indicate that repeated administration of edaravone significantly inhibits lipid peroxidation reaction and protects the brain after ischemia-reperfusion by scavenging radicals. Although the difference in biochemical significance between 16:1 and 18:1 is unknown, inhibition of the increase of both monounsaturated fatty acids in plasma is expected to correlate with decreased oxidative stress, because these monounsaturated fatty acids are formed in compensation for highly unsaturated fatty acids. It is noteworthy that single-dose administration of edaravone was insufficient to inhibit the oxidative damage, and that repeated administration of edaravone for 14 days was required to exert sufficient pharmacological activity. Yamamoto *et al.*^(40)^ also investigated the activity of edaravone on nervous symptoms and motor function (rotarod test, suspension test). Improvements were observed in response to repeated administration of edaravone for 14 days, but were limited or absent after single administration. This may suggest that there is a correlation among nervous symptoms, motor function, and %16:1 and %18:1, and that decrease of these markers—that is, decrease of oxidative damage—is correlated with functional improvements after cerebral infarction.

### Antioxidant activity in humans

#### Antioxidative effects in cerebral infarction

The following four reports show or suggest antioxidative effects of edaravone in patients with cerebral infarction.

**1) Plasma monounsaturated fatty acids.** Unno, Hata, Yamamoto *et al.*^(43)^ measured changes in the plasma concentration of 16:1 as a marker of oxidative stress in acute cerebral embolism patients. Intravenous blood samples were collected at 3–6, 15–18, 24 and 72 h and 7, 14 and 28 days after onset from 10 acute cardioembolic stroke patients for whom the time of onset was clearly known. The plasma levels of 16:1 were significantly increased at all time points prior to 28 days after onset. When the data for the individual patients were examined, the levels were found to be significantly increased in the acute phase, and then fell gradually in the sub-acute phase. The plasma concentration of 16:1 increased to between 2 and 8 times that in the normal controls by around 24 h after onset, and then gradually fell to nearly normal by 4 weeks. The increase of 16:1 level implies an increase in Δ^9^-desaturase activity, and is consistent with the results reported by Yamamoto *et al.*^(40)^ on the plasma levels of monounsaturated fatty acids in the rat MCA occlusion-reperfusion model (see section above). Considering that the inhibition of the increase in the proportion of %16:1 relative to total free fatty acids in the edaravone group in their rat MCA occlusion-reperfusion model was associated with an improvement in neurological symptoms and motor function, it is plausible that the improvement in neurological symptoms seen following the administration of edaravone to humans is a result of inhibition of the increase in %16:1—in other words, inhibition of oxidative damage.

**2) Oxidized low-density lipoprotein.** Uno *et al.*^(44)^ measured oxidized low-density lipoprotein (OxLDL) as a plasma biomarker and evaluated the therapeutic efficacy of edaravone based on the National Institutes of Health Stroke Scale (NIHSS) score in 51 patients with cerebral infarction.

These patients were classified into a group of 24 patients in whom infarction was present in the cortical region (“the cortical group” hereafter) and a group of 27 patients in whom the infarction was present in the basal ganglia or brain stem (“the basal group” hereafter), and the patients were then randomly assigned to an edaravone treatment group (27 patients, 13 from the cortical group and 14 from the basal group) or a non-edaravone treatment group (24 patients, 11 from the cortical group and 13 from the basal group) (Table 5).

The plasma levels of OxLDL on hospital admission were significantly higher in the cortical group than in the basal group (*p*<0.05). The NIHSS scores were also significantly higher in the cortical group than in the basal group (*p*<0.05).

In the cortical group, the plasma OxLDL levels were significantly lower on Days 1 and 3 than at baseline in the patients who received edaravone. These levels gradually fell as time passed, and reached the level of the normal controls, matched for age, on Day 14. The levels were also significantly lower on Days 1 and 3 than in the non-edaravone treatment group. In the non-edaravone treatment group, the levels of OxLDL on Days 1 and 3 were higher than those on Day 0. In the edaravone treatment group, the NIHSS score decreased significantly from a mean of 10.9 on admission to 7.0 at discharge, which correlated with the decrease in plasma OxLDL levels. In the non-edaravone treatment group, on the other hand, the NIHSS score was 12.5 on admission and 13.7 at discharge, and thus showed little change.

In the basal group, the plasma OxLDL level was significantly lower in the edaravone treatment group than in the non-edaravone treatment group on Day 7. In addition, although the NIHSS score in the edaravone treatment group in the basal group decreased somewhat, from 6.4 on admission to 3.6 at discharge, the non-edaravone treatment group showed no change (6.7 on admission and 6.8 at discharge).

It has already been reported that the plasma concentration of OxLDL is a marker of oxidative stress, and it is correlated with infarction volume and/or NIHSS in cerebral infarction in humans.^(45,46)^ The above results show that edaravone is effective in patients with oxidative brain damage, particularly in patients with cortical infarcts, and that OxLDL is an appropriate plasma biomarker for monitoring efficacy.

**3) Circulating neutrophils.** Aizawa *et al.*^(47)^ investigated the effects of edaravone on the intracellular reactive oxygen species of neutrophils and on neutrophil-derived superoxide production induced by phorbol myristate acetate in neutrophils from ischemic stroke patients.

In 21 patients who received edaravone and 19 patients who received ozagrel, blood samples obtained before and after administration were used to measure the level of intracellular reactive oxygen species based on the fluorescence intensity. Edaravone significantly decreased the relative fluorescence units (RFU) from a mean of 36.8 at baseline to 26.8 postdose (*p*<0.0001), whereas ozagrel had no effect (RFU 38.3). In addition, when the level of superoxide produced by neutrophils was measured using chemoluminescence, a significant decrease was found after administration of edaravone (*p*<0.001), while was no change after ozagrel.

Since neutrophils activated by inflammatory response are a potential source of reactive oxygen species, and since edaravone does not react directly with superoxide anion radical and has good cell permeability, these results suggest that edaravone lowers the levels of intracellular reactive oxygen species of neutrophils and inhibits the production of reactive oxygen species by activated neutrophils. These effects are consistent with the clinical efficacy of edaravone in patients with ischemic stroke.

**4) 3-Nitrotyrosine.** Isobe *et al.*^(48)^ measured the concentration of 3-nitrotyrosine (3NT) in cerebrospinal fluid (CSF) following admission in 20 lacunar stroke patients to assess the role of free radicals in lacunar stroke. The mean concentration of 3NT in patients was 10.2 nM, which was significantly higher than the level of 1.4 nM in the controls (*p*<0.0001).

3NT is a powerful neurotoxin^(49)^ formed by reaction of tyrosine with peroxynitrite derived from nitric oxide and superoxide anion radical, and can therefore be considered a biochemical marker of peroxynitrite-induced injury. Based on these results, Isobe *et al.*^(48)^ recommended that edaravone should be administered to patients with lacunar stroke.

#### Investigation of antioxidant effects in ALS

Antioxidant effects of edaravone in ALS patients have been investigated in several studies. In an open-label investigator-initiated study in which edaravone 30 mg was administered once a day for 14 days to patients with ALS, Yoshino and Kimura^(50)^ found that the level of 3NT in CSF was significantly lower at 14 days than at baseline (*n* = 25, *p* = 0.07). In a placebo-controlled double-blind investigator-initiated study of edaravone 30 mg once a day for 20 days, no significant difference was found between the 2 groups in the level of 3NT in CSF (*p* = 0.435). However, in the edaravone group, the level of 3NT at the end of the double-blind period was significantly lower than at baseline (*p* = 0.044).^(51)^ In a Phase 2 open-label study consisting of Cycle 1, in which edaravone 30 or 60 mg was administered for 14 consecutive days followed by a 2-week drug-free period, and Cycle 2 to 6, each consisting of administration for 10 days within a 14-day period followed by a 2-week drug-free period, the level of 3NT at the end of Cycle 6 in CSF was lower than the limit of detection in most of the patients who received edaravone 60 mg.^(52)^

In addition, Nagase *et al.*^(53)^ investigated markers of circulatory oxidative stress and tissue oxidative damage in 26 patients with ALS who received edaravone compared to 55 age-matched healthy controls. In ALS patients who received of edaravone 30 mg per day between 1 and 4 days a week, an increase in uric acid, which is a peroxynitrite scavenger, was confirmed postdose, suggesting that peroxynitrite had been scavenged by edaravone.

## Comparison with Other Antioxidants and Radical Scavengers

**Fig. 8:**
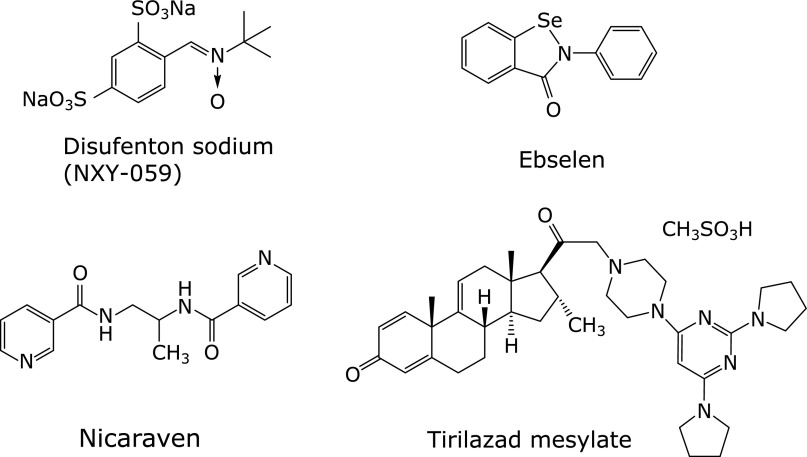
Representative chemical structures of free-radical-eliminating agents and antioxidants.

Here, we discuss the antioxidant activities and physicochemical properties of several representative radical scavengers/antioxidants that have been investigated in clinical studies (up to Phase 3 in some cases) in patients with cerebral infarction and/or subarachnoid hemorrhage (Fig. 8). We also compare their radical-scavenging activities with those of edaravone. It should be noted that at least some of these compounds have intrinsic bioactivities other than direct radical-scavenging/antioxidant effects. Thus, the following discussion does not explain all of the effects that were observed in the clinical studies.

### Disufenton sodium (NXY-059)

**Fig. 9:**
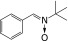
Chemical structure of PBN.

Disufenton is an analogue of α-phenyl-*N*-*tert*-butylnitrone (PBN, Fig. 9), which is used as a spin-trapping agent. It has two sulfonic acid residues on the phenyl moiety, which serve to increase its water solubility. Like PBN, disufenton reacts with free radicals to generate relatively stable spin adducts, thereby blocking free radical-induced injury.^(54,55)^ Disufenton reacts with hydroxyl radical, superoxide anion radical, methyl radical, radicals derived from cyclic ethers such as dioxane and tetrahydrofuran, and radicals derived from alcohols such as methanol and ethanol,^(56)^ and is more reactive than PBN.^(57)^

The calculated partition coefficient (cLogP) value of disufenton is −4.35 (free acids, calculated by Daylight 4.93), which implies extremely low distribution to the lipid phase. This is consistent with the low blood-brain barrier permeability of disufenton.^(58–60)^ Thus, disufenton was suggested to scavenge free radicals at the vascular endothelium, not in the brain parenchyma, and it may react with extracellular reactive oxygen species generated by the interactions of inflammatory cells such as polymorphonuclear leukocytes and macrophages and endothelial cells during reperfusion.^(58,61,62)^ Disufenton decreased the infarction volume in a rat transient MCA occlusion model,^(58,63)^ and was also effective in a rat permanent MCA occlusion model ^(63,64)^ and a guinea pig permanent MCA occlusion model.^(65,66)^ Its activity in the permanent MCA occlusion model may not be consistent with action at the vascular endothelium cell surface,^(60)^ because the concentrations required to suppress infarction volume in the rat transient MCA occlusion model and in the permanent MCA occlusion model are substantially different, and no cell-protective effect was observed *in vitro*.^(67)^ Mechanisms other than free radical scavenging, such as changes in cerebral blood flow,^(68)^ may be important for the action of disufenton.^(54)^

### Ebselen

Ebselen contains selenium, and possesses glutathione peroxidase-like activity based on the different oxidation states of the selenium atom (oxidation numbers: −2 to +4).^(69)^ It inhibits generation of radicals by decomposing hydroperoxides and particularly lipid hydroperoxides, which are free radical precursors, thereby preventing oxidative damage.^(70)^

Ebselen itself shows little radical-scavenging activity, and does not react with DPPH. It is a weaker lipid peroxyl radical scavenger than α-tocopherol, and has little activity to terminate chain reactions; for example, α-tocopherol potently inhibits oxidation of methyl linoleate by radicals derived from azo compounds, whereas ebselen does not inhibit formation of hydroperoxides in the same system.^(71)^ On the other hand, ebselen reacts with superoxide anion radical.^(72)^

Noguchi *et al.*^(71)^ investigated the glutathione peroxidase-like activities of ebselen using a methyl linoleate emulsion. Ebselen inhibited oxidation of the methyl linoleate emulsion in a dose-dependent manner, and decreased hydroperoxide formation by reducing it to the corresponding alcohol. In addition, glutathione promoted ebselen-induced reduction of lipid hydroperoxides to the corresponding alcohols.

Interestingly, the reaction rates of ebselen with linoleic acid hydroperoxide (1.8 × 10^2^ mM^−1^min^−1^) and phosphatidylcholine hydroperoxide (8.8 × 10^2^ mM^−1^min^−1^) are larger than that with hydrogen peroxide (6.6 × 10^1^ mM^−1^min^−1^); ebselen prefers highly lipophilic substrates because it is a lipophilic compound.^(70)^ It also reacts with hydroperoxy phospholipids, hydroperoxy cholesteryl esters and cholesterol hydroperoxide.^(73)^

Ebselen scavenges hydroperoxides via three catalytic cycles, including a typical glutathione peroxidase catalytic cycle. Sarma *et al.*^(74)^ recently discovered that seleninic acid was included in the catalytic cycle as a new intermediate, based on detailed investigation of the reaction mechanism, and proposed the modified catalytic cycle. The properties of the thiol (RSH) in the system greatly affect this reaction. The properties of the hydroperoxides have virtually no effect on the glutathione peroxidase-like activity of ebselen, but those of the thiol have a dramatic effect.^(75)^ The rate-limiting step of the overall reaction is the disproportionation reaction into diselenide and disulfide.^(74,76)^

It is also reported that ebselen readily reacts with peroxynitrite. The reaction rate constant with peroxynitrite is 2.5 × 10^6^ M^−1^s^−1^ (25°C, pH ≥8), which is faster than that of the spontaneous decomposition reaction of peroxynitrite (ONOO^−^)/peroxynitrous acid (ONOOH) at physiological pH. The reaction rate constant with peroxynitrite is fast enough even in the µM concentration range of ebselen. This rate constant is three to four orders of magnitude larger than those for cysteine, ascorbate, and methionine, indicating that ebselen is a good peroxynitrite scavenger.^(77–79)^

The effects of ebselen on lipid peroxidation differ depending on the mechanism of the lipid peroxidation. In an oxidation system of low-density lipoprotein (LDL) with copper (II) chloride, ebselen inhibits lipid peroxidation by scavenging the hydroperoxide that is already present in the LDL and involves in the initial step of lipid peroxidation. Excess ebselen inhibits the lipid peroxidation even in the absence of a thiol. In the case of oxidation of LDLs with azo compounds, which generate peroxy radicals through thermal decomposition, ebselen does not inhibit lipid peroxidation, because the generation of radicals proceeds independently of hydroperoxides. In this case, ebselen reduces the resulting phosphatidylcholine hydroperoxide or cholesteryl ester hydroperoxide into the corresponding alcohol as the major product.^(70,80)^

### Nicaraven (AVS)

Nicaraven is a water-soluble antioxidant with two nicotinamide moieties in its molecule, and primarily scavenges hydroxyl radical. The amide structure is important for this activity.^(81)^ Sugita *et al.*^(82)^ investigated reactions of cumyloxy radical [Ph(CH_3_)_2_CO^•^] with model compounds which mimicked each of the partial structures of nicaraven. The radical-scavenging effects of nicaraven are induced via abstraction of hydrogen atoms of the alkyl groups and the amide groups of nicaraven, namely, donation of a hydrogen atom from the alkyl groups and the amide groups of nicaraven to the cumyloxy radical. The electron-withdrawing effects of the pyridyl group of the nicotinamide are important for hydrogen atom donation, serving to increase the stability of the amidyl radical that are formed, because benzamide and acetamide have virtually no reactivity. Because high reactivity is required to abstract hydrogen atoms from alkyl groups, the radical scavenging activity of nicaraven in the body depends mostly on the two amide bonds; however, the precise mechanism of action is unclear.

The cell membrane permeability of nicaraven is expected to be low because of the two amide bonds; nicaraven forms a seven-membered cyclic conformation in chloroform-*d* through hydrogen bond formation between the two amides, and this has an important impact on the blood-brain barrier permeability.^(81)^ However, it is not certain whether or not the seven-membered cyclic conformation is energetically stable in water, because solvation of nicaraven by water occurs. Akimoto claimed that the permeability of nicaraven into the brain is “fairly good”.^(81)^ However, as the cLogP of nicaraven is 0.077 (calculated by Daylight 4.93), distribution to the brain may be insufficient for activity.

### Tirilazad

Tirilazad mesylate is a steroid-based compound with a 4-(pyrimidin-4-yl)piperazine moiety at the 21-position. It was approved in Austria in 1994 as a therapeutic agent for subarachnoid hemorrahge in males.^(83)^ Tirilazad mesylate inhibits free radical-induced lipid peroxidation through two mechanisms: scavenging hydroxyl, lipid peroxyl, and alkoxyl radicals (a chemical antioxidant effect), and inhibiting the propagation of lipid peroxidation (cell membrane stabilization effect) by reducing the cell membrane fluidity.^(84,85)^ Although tirilazad mesylate is occasionally described as an inhibitor of iron-dependent lipid peroxidation because its effects have often been studied in iron-dependent oxidation models, it also inhibits lipid peroxidation in systems without iron.^(86,87)^ Tirilazad is highly lipophilic (cLogP = 8),^(86)^ and it has a high affinity for the vascular endothelial cell membrane, where it acts first after being administered. Tirilazad has low blood-brain barrier permeability,^(88)^ and distribution to the cerebral parenchyma is limited.^(85)^

The free radical-scavenging activity of tirilazad against various radicals has been investigated.^(89)^ Tirilazad reacts very slowly with peroxyl radicals generated by thermal decomposition of azo compounds and with stable galvinoxyl radical in a dose-dependent manner. These reactions are much slower than those of α-tocopherol, a typical lipid peroxyl radical scavenger. In reactions with azo compound-derived peroxyl radicals, the consumption of tirilazad was inhibited by α-tocopherol. Tirilazad weakly inhibits oxidation of methyl linoleate induced by peroxyl radicals derived from azo compounds in acetonitrile in a dose-dependent manner, with no clear induction period. These results indicate that tirilazad would not be effective to scavenge lipid peroxyl radicals. The reaction rate constant of tirilazad and linoleic acid peroxyl radical in methanol has been measured as being between 9.4 × 10^2^ and 1.8 × 10^3^ M^−1^s^−1^.^(90)^ This low reactivity can be attributed the lack of aromatic amine or reactive phenolic hydroxy groups. The oxidation potential of tirilazad has been reported to be 810 mV.^(91)^

Tirilazad also stabilizes the membrane by restricting the movement of lipid peroxyl radicals and lipid alkoxyl radicals within the cell membrane, thereby inhibiting interactions between the radicals and peroxidizable lipids. The estimated pKa values of tirilazad are 4.2 and 6.5, so that tirilazad should be at least partially protonated *in vivo*. Thus, the positively charged bulky amine moiety would interact ionically with the negatively charged phospholipid head groups in the membrane. The steroid moiety, on the other hand, would interact with fatty acid chains of the membrane. These effects reduce membrane fluidity and also release the compression of the bilayer lipid, thereby increasing the distance between the lipid molecules, and inhibiting free radical propagation.^(86,87)^ Tirilazad at 1.0 mol% (with respect to lipids) significantly increases the viscosity of the head groups in the lipid monomolecular membrane.^(86)^ The hydrophilic hydroperoxyl groups and hydroxy groups are expected to be located in the vicinity of highly polar phospholipids,^(92–94)^ and a decrease in head group fluidity therefore inhibits the movement of these functional groups and reduces the distance between the resulting peroxyl radicals and the C-5 position of the pyrimidine ring that is proposed to be the radical reaction site of tirilazad.^(95)^ Investigation of the effects on straight-chain lecithin monomolecular membranes showed that tirilazad acts to reduce the thickness of the lipid membranes in the liquid phase. This suggests that disorganization readily occurs both within each layer and between layers in the membrane, and that the efficiency of free radical propagation is reduced due to diminished interactions between individual lipid molecules.^(85,95)^

Tirilazad shows a moderate inhibitory activity on soybean phosphatidylcholine liposome membrane peroxidation induced by peroxyl radical. In this case, tirilazad has more potent inhibitory activity on peroxyl radicals attacking from the inside of the membrane than on those outside the membrane. Tirilazad suppressed the consumption of α-tocopherol in the system, and also strongly inhibited peroxidation of the phosphatidylcholine membrane. However, α-tocopherol radical was not reduced by tirilazad, and the mechanism of suppression of the consumption of α-tocopherol is unclear.^(89)^

Accordingly, the antioxidant effects of tirilazad appear to be primarily a result of physicochemical effects on the cell membrane.

### Comparison of the above antioxidants with edaravone

The characteristics of the above antioxidants and edaravone are summarized in Table 6. Although all these antioxidants has been reported to have potent inhibitory activities on lipid peroxidation, ebselen and tirilazad show weak direct radical-scavenging activity. Ebselen inhibits the generation of radicals from lipid hydroperoxide by reducing lipid hydroperoxide. Tirilazad inhibits lipid peroxidation by inducing physicochemical changes in the cell membrane that suppress the interaction between lipids and lipid-derived radicals. Thus, the antioxidant effects of these compounds are based on activities other than *in vivo* radical scavenging. On the other hand, disufenton and nicaraven have direct radical-scavenging activity. Both of these compounds are rather hydrophilic and appear to have poor cell membrane permeability, and are therefore expected to primarily scavenge extracellular radicals.

Various reactive oxygen species are generated in ischemia-reperfusion and inflammation as described in the introduction. Lipids are the most readily oxidizable biological substances, and the peroxyl radicals induced by lipid oxidation are therefore a good target for drugs which act to scavenge/inhibit reactive oxygen species. Edaravone has an appropriate level of lipophilicity for a drug (cLogP: 1.33, calculated by Daylight 4.93), and should be able to enter the membrane by passive diffusion, so that it can scavenge lipid peroxyl radicals.

**Fig. 10:**
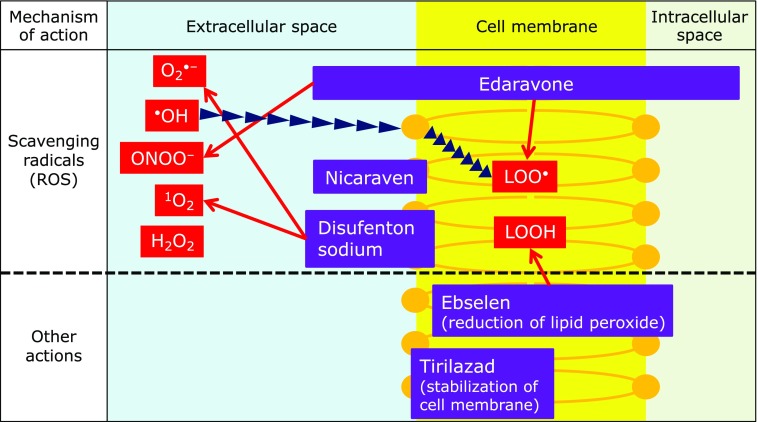
Effects of edaravone and various antioxidants, and mechanisms of action. Reactive oxygen species eliminated by various antioxidants in living organisms are indicated by arrows.

Hydroxyl radical has extremely high reactivity, and immediately react with adjacent organic substances (diffusion controlled reaction), resulting in the generation of other reactive oxygen species such as peroxyl radicals, which also have a direct oxidative effect on biological substances. However, the extremely high reactivity and short life make hydroxyl radical a virtually impossible target for direct scavenging by drugs. In this context, the ability of edaravone to react with a wide range of reactive oxygen species, and its activity to scavenge various reactive oxygen species in either the lipid phase or the water phase represent major advantages, compared to other antioxidants/radical scavengers (Fig. 10).

## Pharmacological Action of Edaravone

### Neuroprotective action

**Fig. 11:**
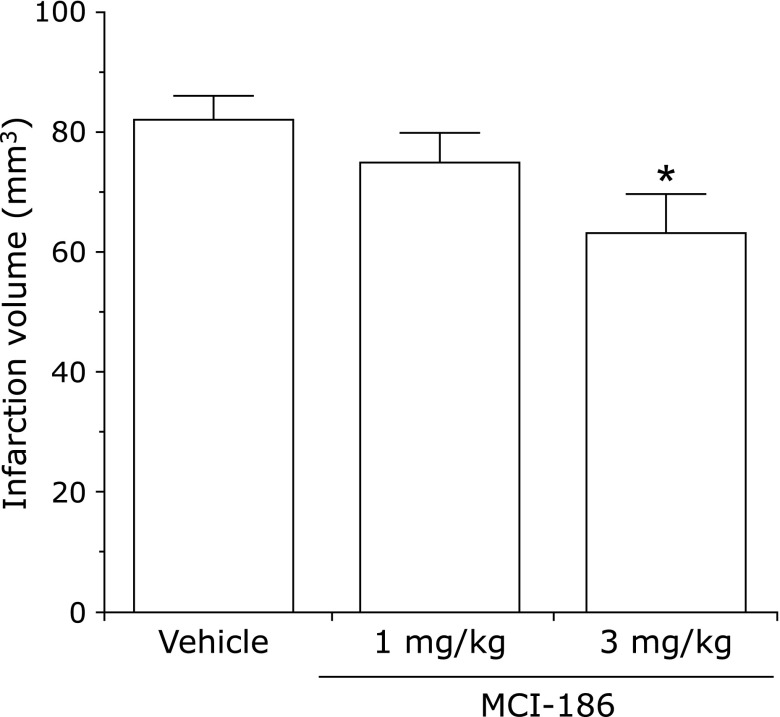
Effects of edaravone (MCI-186) on infarct volume 1 day after distal middle cerebral arterial occlusion. Expressed as the mean ± SE in 10 animals Comparison with the vehicle group: *p<0.05 (Dunnett’s test). (Reprinted from ref 39: ‘Effects of a novel free radical scavenger, MCI-186, on ischemic brain damage in the rat distal middle cerebral artery occlusion model’, J Pharmacol Exp Ther, Kawai H, Nakai H, Suga M, Yuki S, Watanabe T, Saito K, 1997, 281(2), pp. 921–927, reprinted by permission of The American Society for Pharmacology and Experimental Therapeutics, http://www.aspet.org)

**Fig. 12:**
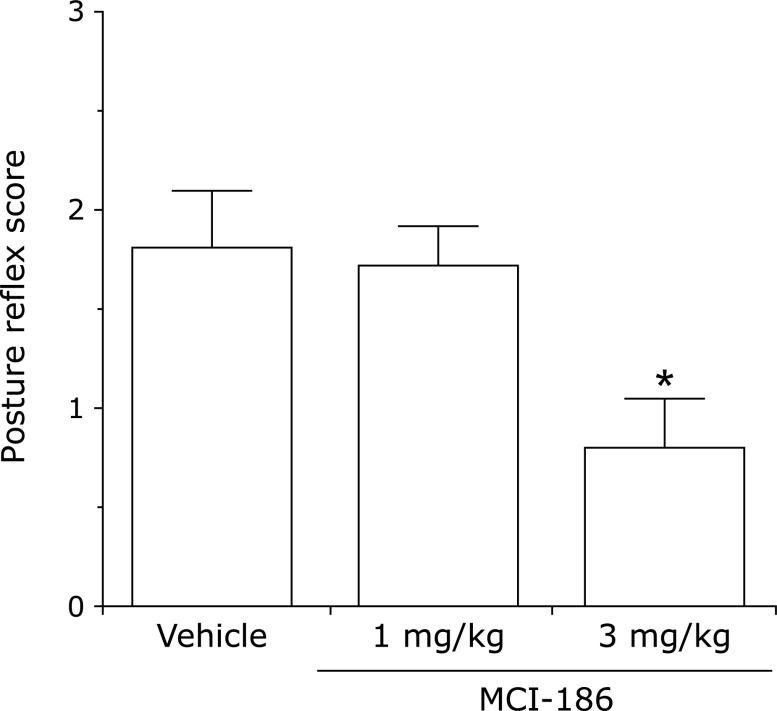
Effects of edaravone (MCI-186) on neuropathy 1 day after distal middle cerebral arterial occlusion. Expressed as the mean ± SE in 10 animals Comparison with the vehicle group: *p<0.05 (nonparametric Dunnett’s test). (Reprinted from ref 39: ‘Effects of a novel free radical scavenger, MCI-186, on ischemic brain damage in the rat distal middle cerebral artery occlusion model’, J Pharmacol Exp Ther, Kawai H, Nakai H, Suga M, Yuki S, Watanabe T, Saito K, 1997, 281(2), pp. 921–927, reprinted by permission of The American Society for Pharmacology and Experimental Therapeutics, http://www.aspet.org)

There are numerous papers describing evaluation of edaravone in stroke models. Abe *et al.*^(96)^ examined its effects against cerebral edema in a middle cerebral arterial occlusion and reperfusion model, termed the “suture model”. In this model, cerebral edema progresses with increasing duration of occlusion up to 6 h, and further damage occurs as a result of reperfusion. Edaravone suppressed edema in both the core and penumbra regions. Local cerebral glucose utilization associated with tissue damage was increased in the penumbra, and edaravone significantly inhibited this increase.^(97)^ Nishi *et al.*^(98)^ reported that edaravone suppressed edema and neurological symptoms in a hemispheric ischemia model. Kawai *et al.*^(39)^ reported alleviatory effects on cerebral infarction volume and neurological symptoms in a rat dMCA occlusion model (Fig. 11 and 12). On the other hand, Yamashita *et al.*^(99)^ investigated the combined use of edaravone and tissue plasminogen activator (tPA) in a spontaneously hypertensive rat reperfusion model. With the suture model, tPA was administered at the time of reperfusion after 4.5 h ischemia, and the mortality rate and the number of animals with severe cerebral hemorrhage were increased in comparison with the group not administered tPA. In addition, increased expression of 4-hydroxynonenal and other lipid oxidation products, as well as matrix metalloprotease-9 (MMP-9), was found in vessels in the penumbra region. When edaravone was administered concomitantly with tPA, the mortality rate and cerebral hemorrhage level were reduced significantly, and vascular lipid oxidation products and MMP-9 expression were also reduced. Lapchak and Zivin^(100)^ reported that there was a therapeutic time window of at least 3 h during which edaravone could be administered effectively to embolism-model rabbits. A protective effect against delayed neuronal death has also been reported.^(101)^

### Action against ALS

Ikeda *et al.*^(17)^ examined the effects of edaravone using the wobbler mouse. The wobbler mouse phenotype resembles the clinical pathology of ALS in terms of protein aggregation and ubiquitination due to over-expression of TAR DNA-binding protein 43 kDa.^(102)^ Edaravone significantly inhibited the decline of grip strength and muscle weight, and suppressed the degeneration of the spinal cord motor neurons at the C5–C6 level after 4 weeks of oral administration, compared to the vehicle group. In addition, intraperitoneal administration for 4 weeks also inhibited the decrease in motor function in a dose-dependent manner.^(103)^ On the other hand, Ito *et al.*^(104)^ reported the effects of edaravone in transgenic mice with the G93A mutation at superoxide dismutase 1 (SOD1). Although no increase in survival time was found, the decline of motor function evaluated with the rotarod and grip strength tests was significantly ameliorated; protection of spinal cord motor neurons and inhibition of SOD1-aggregated protein were also observed. Aoki *et al.*^(105)^ evaluated the effects of edaravone in SOD1 transgenic rat with the H46R mutation. Edaravone or saline was administered by i.v. infusion (1 h infusion per day) for 2 days followed by a 2 drug-free days. In this study, edaravone had no effect on survival duration, but did suppress decrease in motor function.

All the above results suggest that edaravone has protective effects on motor neurons, ameliorates the decline of motor function, and delays progression of symptoms.

Recently, disruption of the blood-brain barrier (BBB)/blood-spinal cord barrier (BSCB) was found in both sporadic ALS patients and SOD1 transgenic mice in the early stage of disease.^(106,107)^ Major causes of the BBB/BSCB impairment include endothelial cell injury and astrocyte degeneration. Furthermore, there have been reports of microglial and astrocytic gliosis, damage to oligodendrocyte precursor cells (OPCs), and demyelination in the brain and spinal cord of ALS patients.^(108)^ Edaravone inhibited oxidative stress-induced damage to vascular endothelium^(32)^ and astrocytes,^(109)^ and improved the differentiation of OPCs to oligodendrocytes.^(110)^ Therefore, edaravone may have protective effects on motor neurons mediated by protection of the vascular endothelium and glial cells.

## Clinical Efficacy of Edaravone

### Efficacy against acute cerebral infarction

After the Phase 1 clinical study of edaravone for acute cerebral infarction in 1987,^(111)^ Phase 2 studies were initiated in 1988,^(112,113)^ and a placebo-controlled Phase 3 study was performed from 1993 to 1996.^(114)^ Here, the results of the Phase 3 placebo-controlled study and a clinical pharmacology study using hydrogen magnetic resonance spectroscopy (^1^H-MRS) are summarized.

#### Placebo-controlled Phase 3 study^(114)^

On the basis of the results of the late Phase 2 study, it was judged that the appropriate dosage and administration method were intravenous administration of two daily doses of 30 mg, and the Phase 3 study was performed targeting neurological symptoms and activity of daily living. On the day of discharge from hospital within 3 months after administration initiation, or at 3 months after administration initiation if the patient remained hospitalized, the functional prognosis was evaluated using the modified Rankin scale, which is in widespread international use. A significant inter-group difference was found by the rank-sum test, and the proportion of entirely symptom-free patients was found to be higher in the edaravone group (E group) than in the placebo group (P group): 22% (27 of 121) and 10% (12 of 120), respectively (Table 7). The outcome data at 3, 6 and 12 months after onset showed that the benefit was sustained (Table 8). When subset analysis was performed for patients treated within 24 h according to instructions by the Japanese regulatory authority during data review, the difference between the two groups was greater than in the analysis of all patients, as shown in Table 9. No significant difference in adverse reaction frequency was found, with the numbers of patients suffering adverse reactions being 9 of 125 and 14 of 125 in the E and P groups, respectively. The most common adverse reactions in the E group were hepatic dysfunction and rash.

#### Clinical pharmacology study using ^1^H-MRS^(115)^

**Fig. 13:**
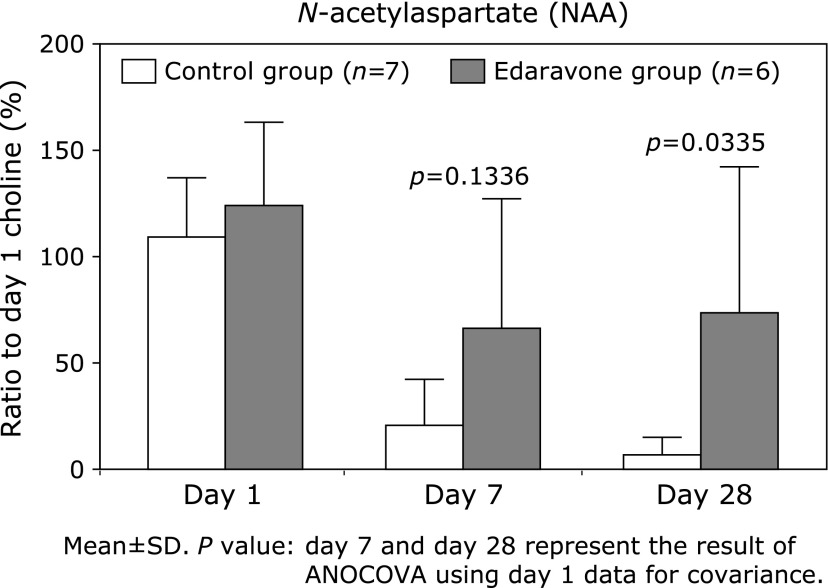
Sequential change of ^1^H-MRS profile.

A clinical pharmacology study was performed from 1993 to 1995, in cerebral infarction patients within 24 h-after onset. The region of interest was defined as the infarct area, and *N*-acetyl aspartate (NAA) was quantified by ^1^H-MRS on day 1, 7 and 28 after onset. NAA is a neuron-specific amino acid derived from brain tissue. It decreases immediately after cerebral infarction onset, and is scarcely detectable in damaged tissue from 24 h after onset. In the control group, NAA decreased with time after infarction onset, and was almost completely eliminated by day 28, whereas in the edaravone group the level on day 28 remained approximately 50% of that immediately after onset (Fig. 13). This demonstrates neuroprotective activity of edaravone, and provides a link between the pharmacological and clinical results.

On the basis of the above findings, edaravone (Radicut injection 30 mg) was approved in 2001, with the indications being neurological symptoms, disability in activities of daily living, and functional impairment associated with acute ischemic stroke, and with administration being initiated within 24 h after the onset of stroke.

Representative results of clinical research performed after approval, and overseas clinical studies, are summarized below.

#### Therapeutic effect of edaravone on severe carotid territory stroke

Edaravone was administered for 14 days to 30 stroke patients with internal carotid artery occlusion and baseline NIHSS scores of at least 15. These patients were compared with a historical control group of 31 similar patients.^(116)^

The infarct volume measured by computed tomography was lower in the edaravone group, at 154 and 241 cm^3^ on day 2 and days 5 to 7, respectively, in comparison with 241 and 321 cm^3^ in the control group. The midline shift was lower in the edaravone group, with means of 3.5 mm on day 2, and 8.2 mm on days 5 to 7, compared with the control group, in which it was 7.7 and 11.5 mm, respectively. The numbers of patients who died within 14 days after onset were 6 (20%) and 14 (45%) in the edaravone and control groups, respectively. Among all patients, only two required no nursing care 8 weeks after onset, and these were both in the edaravone group.

Early-stage edaravone treatment delayed progression of infarction and edema in severe carotid territory stroke patients, and reduced the acute-phase mortality rate. However, no significant improvement in chronic functional outcome in surviving patients was found, and it is therefore concluded that combination therapy of edaravone with antithrombosis and/or thrombolytic agents is required for treating severe stroke.

#### New formulation and dosing regimen in Europe

The protocol followed in Japan involves twice-daily intravenous infusion for up to 14 days. In Europe, however, hospitalization periods for acute conditions are much shorter, so the Japanese protocol was not practical. Therefore, Kaste *et al.*^(117)^ performed a safety, tolerability, and pharmacokinetics study from 2008 to 2010, involving administration of a new formulation of edaravone to acute ischemic stroke patients, with a new dosing regimen of intravenous bolus plus infusion.

The study design was a multicenter, double-blind, placebo-controlled, randomized clinical trial. Study drug or placebo was supplied in 50-ml vials, with an ethanol concentration of 50% (v/v). Two intravenous infusion options were studied: cohort 1 received a loading dose of 0.08 mg/kg followed by a continuous infusion of 0.2 mg/kg/h for 72 h, and cohort 2 received a loading dose of 0.16 mg/kg followed by a continuous infusion of 0.4 mg/kg/h for 72 h. A total of 36 patients were randomized, of whom 12 from cohort 1 and 13 from cohort 2 received edaravone; 11 patients were pooled from cohorts 1 and 2 in a placebo group. Mean NIHSS scores on admission of patients from cohorts 1 and 2 and the placebo group were 5, 5 and 6, respectively.

The results of this study revealed that the dosing regimens are well tolerated. Geometric mean values of edaravone plasma concentration at the end of the infusion in cohorts 1 and 2 were 391 and 1,595 ng/ml, respectively, which are within the putative therapeutic range in humans.

The modified Rankin scale score was evaluated on day 87 (0: no symptoms at all, 1: no significant disability despite symptoms, 2: slight disability, 3: moderate disability, 4: moderately severe disability, 5: severe disability). The numbers of patients with the respective scores in cohort 1 were 1, 5, 1, 5, 0 and 0 (*n* = 12); those in cohort 2 were 3, 6, 3, 1, 0 and 0 (*n* = 13); and those in the placebo group were 0, 5, 3, 3, 0 and 0 (*n* = 11).

The primary objective of this study, namely, to establish safety and tolerability of the new formulation and dosing regimen, was achieved.

### Efficacy against ALS

One Phase 2 study and four Phase 3 studies were conducted. The Phase 2 study was an open-label study with ALS patients, initiated in 2001.^(52)^ Of the Phase 3 studies, one was a placebo-controlled study with ALS patients not requiring nursing, initiated in 2006,^(118)^ followed by an extension study. The next study was an exploratory study with more advanced-stage ALS patients. A subsequent Phase 3 study was a placebo-controlled replicate study with ALS patients, initiated in 2011.^(119)^ The results of the two Phase 3 studies conducted from 2006 and 2011 are summarized below.

In the Phase 2 study, the change in Revised ALS Functional Rating Scale (ALSFRS-R) score was significantly less during the 6-month treatment period with 60 mg edaravone than during the preceding 6-month period before initiation of edaravone administration (mean difference with standard deviation (SD): 2.4 ± 3.5 points; Wilcoxon signed rank test; *p* = 0.039). The Phase 3 study to confirm the efficacy observed in Phase 2 was initiated in 2006. The inclusion criteria were as follows: (i) age: 20 to 75; (ii) definite, probable, or probable laboratory-supported ALS according to the revised El Escorial diagnostic criteria; (iii) force vital capacity (FVC) at least 70%; (iv) disease duration of 3 years or less; (v) change in ALSFRS-R score during the 12-week pre-observation of −1 to −4; and (vi) independent living status, grade 1 or 2 according to the Japan ALS Severity Classification. The study design included a 12-week pre-observation period, followed by a 24-week double-blind treatment period (6 cycles). In cycle 1, the study drug was administered for 14 consecutive days followed by a 2-week drug-free period. In cycle 2 and thereafter, the study drug was administered for 10 days within a 14-day period, followed by a 2-week drug-free period. The changes in ALSFRS-R scores during the 24-week treatment (primary outcome) were −6.35 ± 0.84 in the placebo group (*n* = 99) and −5.70 ± 0.85 in the edaravone group (*n* = 100). The difference between the two groups was 0.65 ± 0.78 (*p* = 0.411). The incidences of serious adverse events were 23.1% and 17.6% in the placebo and edaravone groups, respectively. The common serious adverse events were dysphagia and gait disturbance, which were due to ALS.

Post-hoc analysis suggested a favorable trend among patients meeting the following criteria.

1) scores of ≥2 points on all items of ALSFRS-R,

2) %FVC of ≥80% at baseline,

3) definite or probable ALS (El Escorial/revised Airlie House criteria),

4) disease duration of ≤2 years.

Since post-hoc analyses of clinical studies have limitations in interpretability, a further Phase 3 study was conducted from 2011, with a similar design and the above criteria.

The change in ALSFRS-R scores during the 24-week treatment (primary outcome) was −7.50 ± 0.66 in the placebo group and −5.01 ± 0.64 in the edaravone group. The least-squares mean value of difference between groups was 2.49 ± 0.76 (*p* = 0.0013; Table 10) in favour of edaravone. Change in %FVC showed a positive trend with edaravone, although the difference was not statistically significant (*p* = 0.0942). Modified Norris Scale and the 40-item Amyotrophic Lateral Sclerosis Assessment Questionnaire (ALSAQ-40) showed statistically significant levels of difference from placebo (*p* = 0.0393; *p* = 0.0309, respectively). The incidences of adverse events were 83.8% and 84.1% in the placebo and edaravone groups, respectively. No significant differences in either adverse events or serious adverse events were found between these groups.

## Conclusions

We have summarized the radical-scavenging and antioxidant activities of edaravone in comparison of other radical scavengers and antioxidants, together with the activities of edaravone in cerebral infarction and ALS models, and its clinical effects in humans. Edaravone shows marked scavenging effects on various reactive oxygen species and radicals including peroxynitrite. Approximately 50% of edaravone is present as the anion under physiological conditions, and the anion scavenges radicals by donating one electron to a radical. As a result, edaravone itself is converted to OPB irrespective of the radical species. Non-ionized edaravone can passively diffuse through biological membranes, and thus edaravone is capable of scavenging radicals in both aqueous and lipid phases. It is also considered that edaravone exerts its antioxidant activity cooperatively with endogenous antioxidants such as ascorbic acid and vitamin E. Based on these characteristics, edaravone has clinically used in Japan since 2001 as a therapeutic agent for acute cerebral infarction, and it has been established as a standard therapy for acute ischemic stroke in Japan to date. In addition, Japanese manufacturing and marketing approval of edaravone for treatment of ALS was given in 2015, for suppression of progression of functional disorders. Then in 2017, the U.S. Food and Drug Administration (https://www.accessdata.fda.gov/drugsatfda_docs/label/2017/209176lbl.pdf, cited June 13, 2017) also approved edaravone for treatment of patients with ALS.

There have been numerous reports on edaravone. Short summaries of some representative publications are given below.

## Nonclinical Studies

- Yamamoto, Y, *et al.* Antioxidant activity of 3-methyl-1-phenyl-2-pyrazolin-5-one. *Redox Rep* 1996; **2**: 333–338. (ref 31) The electron transfer of edaravone anion to lipid peroxyl radical inhibits lipid peroxidation. Synergistic inhibition of peroxidation with ascorbic acid or α-tocopherol in artificial lipid membrane system.
- Fujisawa A, Yamamoto Y. Edaravone, a potent free radical scavenger, reacts with peroxynitrite to produce predominantly 4-NO-edaravone. *Redox Rep* 2016; **21**: 98–103. (ref 37) The physicochemical studies of the reactivity of edaravone and peroxynitrite, and comparison with uric acid, which scavenge peroxynitrite in the body.
- Yamamoto Y, *et al.* Repeated edaravone treatment reduces oxidative cell damage in rat brain induced by middle cerebral artery occlusion. *Redox Rep* 2009; **14**: 251–258. (ref 40) *In vivo* studies of inhibition of lipid peroxidation, and effects on neurological symptoms and motor function, in a transient MCA occlusion model, using plasma 16:1 and 18:1 as markers.
- Watanabe T, *et al.* Preventive effect of MCI-186 on 15-HPETE induced vascular endothelial cell injury *in vitro*. *Prostagland Leukotr Essent Fatty Acids* 1988; **33**: 81–87. (ref 32) Edaravone inhibited vascular endothelial damage induced by 15-HPETE.
- Abe K, *et al.* Strong attenuation of ischemic and post ischemic brain edema in rats by a novel free radical scavenger. *Stroke* 1988; **19**: 480–485. (ref 96) Edaravone suppressed an increase in water content (cerebral edema) in a middle cerebral artery occlusion-reperfusion model.
- Kawai H, *et al.* Effects of a novel free radical scavenger, MCI-186, on ischemic brain damage in the rat distal middle cerebral artery occlusion model. *J Pharmacol Exp Ther* 1997; **281**: 921–927. (ref 39) The detection of OPB, generated from edaravone as a radical scavenging product, in the ischemic brains of rats. Edaravone also suppressed cerebral infarction and alleviated neurological symptoms.
- Ikeda K, *et al.* Treatment of wobbler mice with free radical scavenger. Molecular mechanisms and therapeutics of amyotrophic lateral sclerosis. *Int Congress Series* 2001; **1221**: 335–339. (ref 17) The first published report of the efficacy of edaravone in the wobbler mouse.
- Lee BJ, *et al.* Edaravone, a free radical scavenger, protects components of the neurovascular unit against oxidative stress *in vitro*. *Brain Res* 2010; **1307**: 22–27. (ref 109) The protective effects of edaravone against oxidative stress in the neurovascular unit.

## Clinical Studies

- The Edaravone Acute Brain Infarction Study Group (chair: Otomo E). Effect of a novel free radical scavenger, edaravone (MCI-186), on acute brain infarction: Randomized, placebo-controlled, double-blind study at multicenters. *Cerebrovasc Dis* 2003; **15**: 222–229. (ref 114) The results of the Phase 3 study with acute cerebral infarction patients, demonstrating efficacy in terms of the modified Rankin Scale.
- Houkin K, *et al.* Neuroprotective effect of the free radical scavenger MCI-186 in patients with cerebral infarction: clinical evaluation using magnetic resonance imaging and spectroscopy. *J Stroke Cerebrovasc Dis* 1998; **7**: 315–322. (ref 115) The results of a clinical pharmacology study with acute cerebral infarction patients, which indicates efficacy on the basis of magnetic resonance imaging and spectroscopy.
- Yoshino H, Kimura A. Investigation of the therapeutic effects of edaravone, a free radical scavenger, on amyotrophic lateral sclerosis (Phase II study). *Amyotroph Lateral Scler* 2006; **7**: 247–251. (ref 52) The results of the Phase 2 open study with ALS patients, which indicates efficacy (ALSFRS-R) and reduction of oxidative stress marker (3NT) levels.
- Nagase M, *et al.* Increased oxidative stress in patients with amyotrophic lateral sclerosis and the effect of edaravone administration. *Redox Rep* 2016; **21**: 104–112. (ref 53) Edaravone treatment increased plasma levels of uric acid in patients with ALS, suggesting that edaravone is an effective scavenger of peroxynitrite *in vivo*.
- The Writing Group on behalf of the Edaravone (MCI-186) ALS 19 Study Group. Safety and efficacy of edaravone in well defined patients with amyotrophic lateral sclerosis: a randomised, double-blind, placebo-controlled trial. *Lancet Neurol* 2017; **16**: 505–512. (ref 119) The results of the second Phase 3 study with ALS patients, which indicates efficacy with respect to ALSFRS-R.

## Figures and Tables

**Table 1:** Physicochemical properties of edaravone

Chemical name	3-methyl-1-phenyl-2-pyrazolin-5-one

Code No.	MCI-186

Molecular formula	C_10_H_10_N_2_O

Molecular weight	174.2

Description	White to slightly yellowish white crystals or powder.
	Freely soluble in methanol, ethanol (99.5), or acetic acid (100); slightly soluble in water or diethyl ether.
	Solubility in water: 1.85 ± 0.15 mg/ml^(19)^

Melting point	127–131°C

Distribution coefficient (Log P)	1.24 (1-octanol/buffer, pH 6.0) 0.45 (1-octanol/buffer, pH 8.0)

Acid dissociation constant (pKa)	7.0

**Table 2:** Oxidation potential of edaravone

Oxidation potential^†^	pH	Reference
483 mV	7	21
480 mV	7.8	21
422 mV	10.0–10.1	22

^†^Counter electrode: Ag^+^/AgCl

**Table 3:** Rate constants of reactions between edaravone and various radicals

Radical	Reaction rate constant	Conditions	Reference
^•^OH	3.0 × 10^10^ M^−1^s^−1^	electron paramagnetic resonance (EPR) – spin trapping method with DMPO, 0.1 M phosphate buffer (pH 7.0)	24
	8.5 ± 0.4 × 10^9^ M^−1^ s^−1^	pulse radiolysis, H_2_O, (pH 7.0)	25

Cl_3_COO^•^	5.0 ± 0.2 × 10^8^ M^−1^ s^−1^	pulse radiolysis, *iso*-PrOH:H_2_O:CCl_4_ = 48:48:4 (% v/v), (pH 9.7)	25

N_3_^•^	5.8 ± 0.3 × 10^9^ M^−1^ s^−1^	pulse radiolysis, H_2_O, (pH 9)	25

SO_4_^•−^	6.0 ± 0.5 × 10^8^ M^−1^ s^−1^	pulse radiolysis, H_2_O, (pH 9)	25

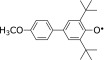	6.28 × 10^1^ M^−1^s^−1^(pH 6)	stopped-flow spectrophotometer, pH 6–8 (H_2_O/MeOH)	26
	1.08 × 10^2^ M^−1^s^−1^(pH 8)		

**Table 4:** Relative rate constants for elimination reactions with various radicals

	^•^OH	O_2_^•−^	*tert*-BuOO^•^	Me^•^	^1^O_2_
Edaravone	1	1	1	1	1
Uric acid	0.0311	3.63	~0	~0	0.084
Glutathione	0.055	0.381	0.105	0.0664	34.1
Trolox	0.829	0.717	~0	0.157	2.26

Prepared from reference 30.

**Table 5:** Effects of edaravone on oxidized low-density lipoprotein (OxLDL)

	Cortical group	Basal group
Edaravone treatment group	– number of patients: 13	– number of patients: 14
	– plasma OxLDL	– plasma OxLDL
	baseline: 0.219 ± 0.016 ng/µg of apoB protein on day 1 and 3, significantly lower than baseline day 3: 0.177 ± 0.024 ng/µg of apoB protein on day 1 and 3, significantly lower than that in non-edaravone group	baseline: 0.156 ± 0.013 ng/µg of apoB protein on day 7, significantly lower than that in the non-edaravone group
	– NIHSS score	– NIHSS score
	at hospitalization (average): 10.9 ± 2.5	at hospitalization (average): 6.4 ± 2.3
	→ at discharge (average): 7.0 ± 1.2 (significant decrease, *p*<0.05)	→ at discharge (average) : 3.6 ± 1.2

Non-edaravone treatment group	– number of patients: 11	– number of patients: 13
	– plasma OxLDL	– plasma OxLDL
	baseline: 0.221 ± 0.028 ng/µg of apoB protein on days 1 and 3, higher than on day 0	baseline: 0.157 ± 0.028 ng/µg of apoB protein
	day 3: 0.219 ± 0.026 ng/µg of apoB protein	
	– NIHSS score	– NIHSS score
	at hospitalization (average): 12.5 ± 2.8	at hospitalization (average): 6.7 ± 3.7
	→ at discharge (average): 13.7 ± 4.2	→ at discharge (average): 6.8 ± 3.6

**Table 6:** Mechanisms of action of edaravone and various antioxidants

Antioxidant	Scavenging activities against free radicals and peroxynitrite	Mechanism of antioxidant effects
Disufenton	– reacts with hydroxyl radicals and alcohol- and/or ether-derived radicals to form adducts.	– possesses low BBB permeability, does not migrate into the cell, and eliminates radical species derived from vascular endothelium extracellularly.

Ebselen	– reacts with superoxide anion radical and peroxynitrile, but not with peroxyl radicals or other similar radicals.	– reduces radical precursors by reducing lipid peroxides to the corresponding alcohols by glutathione peroxidase-like action in collaboration with thiols *in vivo*.

Nicaraven	– eliminates hydroxyl radicals and the like by donating a hydrogen atom from the amide structure.	– based on its molecular structure and lipophilicity, it is presumed that its brain permeability is not high, but details are unknown.

Tirilazad	– weakly reactive with radicals	– migrates to the cell membrane of the vascular endothelium, decreases the interaction between lipid molecules by reducing the membrane fluidity of cells, and suppresses the propagation of lipid peroxidation by radical chain reaction.
		– suppresses membrane peroxidation by a synergistic effect with vitamin E in the membrane.

Edaravone	– scavenges broad range of radical species and peroxynitrite, but does not react with superoxide anion radicals or hydroperoxides.	– readily enters biological membrane and distribute both plasma and tissue.
	– its anionic form has high radical scavenging activity.	– suppresses peroxidation by synergistic effects with α-tocopherol and ascorbic acid in the membrane.

**Table 7:** The modified Rankin Scale scores assessed at discharge within 3 months or at 3 months after onset

Grade	0	1	2	3	4	5	Death	
Edaravone (*n* = 125)	27	36	29	12	10	7	4	*p* = 0.0382*
Placebo (*n* = 125)	12	35	40	12	15	6	5	

Grade: 0, no symptoms at all; 1, no significant disability despite symptoms; 2, slight disability; 3, moderate disability; 4, moderately severe disability; 5, severe disability. *Wilcoxon’s rank sum test.

**Table 8:** The modified Rankin Scale scores assessed at 3 months, 6 months and 12 months after onset

Grade		0	1	2	3	4	5	Death	
3 months	edaravone (*n* = 115)	26	34	24	10	9	7	5	*p* = 0.0481*
	placebo (*n* = 113)	10	39	26	11	14	7	6	

6 months	edaravone (*n* = 105)	27	35	15	11	3	8	6	*p* = 0.0112*
	placebo (*n* = 103)	9	37	23	10	9	7	8	

12 months	edaravone (*n* = 100)	27	31	14	8	4	6	10	*p* = 0.0248*
	placebo (*n* = 94)	8	35	19	7	11	5	9	

Grade: 0, no symptoms at all; 1, no significant disability despite symptoms; 2, slight disability; 3, moderate disability; 4, moderately severe disability; 5, severe disability. *Wilcoxon’s rank sum test.

**Table 9:** The modified Rankin Scale scores assessed at discharge within 3 months or at 3 months after onset, in patients treated within 24 h after onset.

Grade	0	1	2	3	4	5	Death	
Edaravone (*n* = 42)	14	10	8	5	2	2	1	*p* = 0.0001*
Placebo (*n* = 39)	1	6	13	3	8	4	4	

Grade: 0, no symptoms at all; 1, no significant disability despite symptoms; 2, slight disability; 3, moderate disability; 4, moderately severe disability; 5, severe disability. *Wilcoxon’s rank sum test.

**Table 10:** Differences in ALSFRS-R scores between Cycle 1 (Baseline) and Cycle 6 (LOCF)

Treatment group	Number of subjects	Adjusted mean change from baseline		Between-group differences in adjusted mean	
		LS mean ± SE		LS mean ± SE (95% CI)	*p* value^†^
Placebo	66	−7.50 ± 0.66		2.49 ± 0.76 (0.99, 3.98)	*p* = 0.0013
Edaravone	68	−5.01 ± 0.64			

^†^Compared between treatment groups using an analysis of variance (ANOVA) with treatment group and three dynamic allocation factors. For patients with missing values at the end of cycle 6, data were imputed by the LOCF method, provided that they had completed at least to cycle 3. ALSFRS-R, Revised Amyotrophic Lateral Sclerosis Functional Rating Scale; CI, confidence interval; LOCF, last observation carried forward; LS, least squares; SE, standard error.

## Abbreviations

16:1: palmitoleic acid
18:1: oleic acid
%16:1: percentage of plasma palmitoleic acid in total free fatty acids
%18:1: percentage of plasma oleic acid in total free fatty acids
ALS: amyotrophic lateral sclerosis
ALSFRS-R: Revised ALS Functional Rating Scale
ArO^•^: 2,5-di-tert-butyl-4-(4-methoxyphenyl)phenoxyl
BBB: blood-brain barrier
BPOH: 4-hydroxy-4-(3-methyl-5-oxo-1-phenyl-2-pyrazolin-4-yl)-3-methyl-1-phenyl-2-pyrazolin-5-one
BSCB: blood-spinal cord barrier
cLogP: calculated partition coefficient
CSF: cerebrospinal fluid
DPPH: 1,1-diphenyl-2-picrylhydrazyl
ESR: electron spin resonance
FVC: force vital capacity
^1^H-MRS: hydrogen magnetic resonance spectroscopy
15-HPETE: 15-hydroperoxy-eicosatetraenoic acid
IC_25_: 25% inhibitory concentration
IC_50_: 50% inhibitory concentration
LDL: low-density lipoprotein
MCA: middle cerebral artery
MMP-9: matrix metalloprotease-9
NAA: *N*-acetyl aspartate
NIHSS: National Institutes of Health Stroke Scale
4-NO-edaravone: 3-methyl-4-nitroso-1-phenyl-pyrazolin-5-one
4-NO_2_-edaravone: 3-methyl-4-nitro-1-phenyl-pyrazolin-5-one
3NT: 3-nitrotyrosine
ONOO^−^: peroxynitrite
ONOOH: peroxynitrous acid
OPB: 2-oxo-3-(phenylhydrazono)butanoic acid
OPCs: oligodendrocyte precursor cells
OxLDL: oxidized low-density lipoprotein
4-oxoedaravone: 3-methyl-1-phenyl-2-pyrazolin-4,5-dione
pKa: acid dissociation constant
PBN: α-phenyl-*N*-*tert*-butylnitrone
RFU: relative fluorescence units
SIN-1: 3-(4-morpholinyl)sydnonimine hydrochloride
SOD1: superoxide dismutase 1
TP: 4,4-bis-(3-methyl-5-oxo-1-phenyl-2-pyrazolin-4-yl)-3-methyl-1-phenyl-2-pyrazolin-5-one
tPA: tissue plasminogen activator
